# Transcriptional Factor DLX3 Promotes the Gene Expression of Enamel Matrix Proteins during Amelogenesis

**DOI:** 10.1371/journal.pone.0121288

**Published:** 2015-03-27

**Authors:** Zhichun Zhang, Hua Tian, Ping Lv, Weiping Wang, Zhuqing Jia, Sainan Wang, Chunyan Zhou, Xuejun Gao

**Affiliations:** 1 Department of Cariology and Endodontology, School and Hospital of Stomatology, Peking University, Beijing, PR China; 2 Department of Biochemistry and Molecular Biology, School of Basic Medical Sciences, Peking University, Beijing, PR China; Baylor College of Medicine, UNITED STATES

## Abstract

Mutation of distal-less homeobox 3 (DLX3) is responsible for human tricho-dento-osseous syndrome (TDO) with amelogenesis imperfecta, indicating a crucial role of DLX3 in amelogenesis. However, the expression pattern of DLX3 and its specific function in amelogenesis remain largely unknown. The aim of this study was to investigate the effects of DLX3 on enamel matrix protein (EMP) genes. By immunohistochemistry assays of mouse tooth germs, stronger immunostaining of DLX3 protein was identified in ameloblasts in the secretory stage than in the pre-secretory and maturation stages, and the same pattern was found for *Dlx3* mRNA using Realtime PCR. In a mouse ameloblast cell lineage, forced expression of DLX3 up-regulated the expression of the EMP genes *Amelx*, *Enam*, *Klk4*, and *Odam*, whereas knockdown of DLX3 down-regulated these four EMP genes. Further, bioinformatics, chromatin immunoprecipitation, and luciferase assays revealed that DLX3 transactivated *Enam*, *Amelx*, and *Odam* through direct binding to their enhancer regions. Particularly, over-expression of mutant-DLX3 (c.571_574delGGGG, responsible for TDO) inhibited the activation function of DLX3 on expression levels and promoter activities of the *Enam*, *Amelx*, and *Odam* genes. Together, our data show that DLX3 promotes the expression of the EMP genes *Amelx*, *Enam*, *Klk4*, and *Odam* in amelogenesis, while mutant-DLX3 disrupts this regulatory function, thus providing insights into the molecular mechanisms underlying the enamel defects of TDO disease.

## Introduction

Mutation of distal-less homeobox 3 (DLX3) gene, a member of the distal-less homeodomain family (DLX1-6), is responsible for a human autosomal-dominant disease, tricho-dento-osseous syndrome (TDO; OMIM 190320) [1,2]. The most common mutation form is a 4-bp deletion (c.571_574delGGGG) in coding sequence of DLX3. TDO-affected individuals with this mutation have defects in tooth, hair, and bone, and the most penetrant phenotype features are dental findings of enamel hypoplasia and hypomaturation with taurodontism (elongation of the dental pulp chamber), suggesting a specific role of DLX3 in amelogenesis [3]. The process of amelogenesis is divided into three main phases: the pre-secretory, secretory, and maturation stages. During this process, the sequential expression and secretion of enamel matrix proteins (EMPs) are critical and considered to be co-regulated by transcriptional factors, cytokines, growth factors, and signaling molecules [4,5]. Since the hypoplastic and hypomaturation enamel defects of TDO are comparable to those caused by mutations of EMP genes such as *AMELX*, *AMBN*, *ENAM*, *MMP20*, and *KLK4* [6–8], a possible link between *DLX3* and EMP genes is indicated.

DLX3 is expressed in the placenta during early embryonic development, and is later found in skin and bone, as well as tissues derived from epithelial-mesenchymal interactions, including dental epithelium and mesenchyme [9,10]. As is regarded to be a transcriptional activator, DLX3 is composed of a DNA-binding homeodomain and two transactivation domains separately located in the N-terminal region or just downstream of the homeodomain [11]. *Ex vivo* examination of osteoblastic and keratinocyte cell lines have shown that the mutant DLX3 responsible for TDO (DLX3^TDO^) exerts a dominant-negative effect on the normal function of wild-type DLX3, which might lead to the abnormal phenotypes [12].

Studies have strongly suggested the pivotal role of DLX3 in controlling matrix deposition and biomineralization. In osteoprogenitor cells, DLX3 promotes the expression of bone matrix proteins such as type 1 collagen, bone sialoprotein, osteocalcin, and alkaline phosphatase [13]. In particular, chromatin immunoprecipitation (ChIP) assays have confirmed that osteocalcin is directly regulated by DLX3 [14]. During dentin development, mutant DLX3 (containing a 4-bp deletion) in transgenic mice has been reported to disrupt odontoblast cytodifferentiation and lead to odontoblast apoptosis [15]. Furthermore, *ex vivo* studies in odontoblasts have established a mechanistic link between DLX3 and a major dentin matrix protein, DSPP [16]. In addition, DLX3 has been shown to participate in the odontoblastic and osteogenic differentiation process of dental-derived cells [17,18].

In dental enamel, also an important mineralized tissue, DLX3 expression has been found in the pre-secretory ameloblasts of mouse molars [19]. However, the spatio-temporal expression pattern of DLX3 and its exact role in amelogenesis remain largely unknown. In the current study, we characterized the expression pattern of DLX3 in amelogenesis, and analyzed the effects of DLX3 on EMP genes *in vitro*. We found that in amelogenesis, DLX3 plays crucial roles through up-regulating the EMP genes *Amelx*, *Enam*, *Klk4*, and *Odam*, and that mutant-DLX3 disrupts this regulatory function.

## Materials and Methods

### Animals and ethics statement

This study was carried out in strict accordance with the National Institutes of Health Guide for the Care and Use of Laboratory Animals (National Resource Council). All protocols were approved by the Animal Care and Use Committee of Peking University (Permit number: LA 2010–066). Neonatal ICR mice (postnatal days 1, 3, 7, and 14) in the postprandial state were anesthetized with 5 mg/100 g body weight of sodium pentobarbital, and all reasonable efforts were made to ameliorate suffering. For immunochemistry and real-time RT-PCR assay, tooth germs of the first mandibular molars were dissected under a microscope (Zeiss Stemi 2000-C, Germany).

### Cell culture

The mouse ameloblast-like cell lineage, LS8, was a gift from Dr. Malcolm Snead (USC, Los Angeles, CA, USA) [20]. The LS8 cells were maintained in Dulbecco’s modified Eagle’s medium (DMEM; Gibco BRL, Gaithersburg, MD, USA) containing 10% (v/v) fetal bovine serum, penicillin (100 U/ml), and streptomycin (100 μg/ml) in a 37°C, 5% CO_2_ incubator, and passaged just before reaching confluence.

### Plasmid construction

The pCI-neo-V5DLX3^WT^ plasmid, expressing mouse DLX3 under a constitutive CMV promoter, was kindly provided by Dr. Maria Morasso (Developmental Skin Biology Section, NIAMS-NIH, DC, USA). The pCI-neo-V5DLX3^TDO^ plasmid (with the TDO mutation, c.571_574delGGGG) was constructed by GeneChem Technology (Shanghai, China). Constructs of pGL*Enam-E1*, pGL*Enam-E2*, pGL*Amelx-E2*, pGL*Klk4-E1*, and pGL*Odam-E2* were made by cloning the amplified PCR products of the corresponding enhancer regions (with potential DLX3 binding sites) into the pGL3-Promoter Luciferase Reporter Vector (Promega, Madison, WI, USA). Mutations of the potential DLX3 binding sites (T*AA*TT changed to T*GG*TT) on pGL*Enam-E1*, pGL*Amelx-E2*, and pGL*Odam-E2* were performed by TransGen Tech (Beijing, China), generating the constructs Mut pGL*Enam-E1*, Mut pGL*Amelx-E2*, and Mut pGL*Odam-E2*. The mutations were verified by DNA sequencing.

### DLX3 immunohistochemistry

Dissected mouse tooth germs were fixed in 4% paraformaldehyde overnight at 4°C, then decalcified in 10% ethylenediaminetetraacetic acid, dehydrated in a graded series of ethanol, and embedded in paraffin. Serial sections were cut at 5 μm, and then immunohistochemistry was performed with the SP Histostain-Plus kit (ZSGB-BIO, China) based on the instructions, as described previously [21]. Briefly, the sections were deparaffinized, rehydrated, treated with citrate buffer (10 mM, pH 6.0), and microwaved. Then, 3% H_2_O_2_ was added to inactivate endogenous peroxidase, and 10% goat serum was used for blocking. After that, the sections were incubated with anti-DLX3 antibody (code: ab64953, Abcam, Cambridge, MA, USA) overnight at 4°C, or with non-relevant rabbit immunoglobulins as negative control. Goat anti-rabbit secondary antibody (ZSGB-BIO) and diaminobenzidine (Sigma, St. Louis, MO, USA) were then applied, and the slices were visualized under a light microscope (DMRB, Leica, Germany). Using the Image-Pro Plus software (version 6) (Media Cybernetics, USA), the mean optical density of the ameloblasts region [integrated optical density (IOD)/unit area] was determined, which represents the immunoreactivity of DLX3 protein within ameloblast cells.

### Plasmid transfection

LS8 cells were seeded at a density of 2×10^4^ cells per cm^2^ and cultured overnight. For transfection, the VigoFect reagent (Vigorous Biotech, Beijing, China) and plasmid DNA were separately diluted in opti-DMEM (Gibco BRL) for 5 min, mixed at room temperature for 15 min, and then added to the cultures following the manufacturer’s instructions. After 6 h of transfection, the medium was replaced. Cells were harvested and analyzed at 36 or 48 h post-transfection.

### RNA interference

Three different nucleotides (20–23 nt, named *Dlx3* siRNA #1, #2, #3) targeting mouse *Dlx3* mRNA (NM_005220) were produced by Sigma and tested for silencing. A nonspecific siRNA (NS siRNA; Sigma) was used as control. LS8 cells were seeded and cultured until sub-confluence. *Dlx3* siRNA or NS siRNA was diluted in DMEM to a final concentration of 50 nM, combined with the Lipofectamine 2000 reagent (Invitrogen, Carlsbad, CA, USA), incubated at room temperature for 20 min, and then added to the cultures. The medium was replaced 8 h later, and total RNA and protein were extracted at 36 or 48 h post-transfection for further analysis. The sequences of the siRNAs were as follows: *Dlx3* siRNA (#1), GUCACUGACCUGGGCUAUUdTdT, AAUAGCCCAGGUCAGUGACdTdT; *Dlx3* siRNA (#2), CGAACGAUCUACUCCAGCUdTdT, AGCUGGAGUAGAUCGUUCGdTdT; *Dlx3* siRNA (#3), GUGACUCCAUGG CCUGCAAdTdT, UUGCAGGCCAUGGAGUCACdTdT; NS siRNA, UUCUCCGAACGUGUCACGUTT, ACGUGACACGUUCGGAGAATT.

### Western blot

LS8 cells were washed twice with PBS, and then lysed in modified radioimmunoprecipitation assay lysis buffer (Beyotime, Beijing, China) containing 1 mM phenylmethylsulfonyl fluoride. Protein concentration was quantified with the bicinchoninic acid protein assay (Pierce, Rockford, IL, USA). Aliquots of 50 μg protein extract per sample were subjected to 10% sodium dodecyl sulfate-polyacrylamide gel electrophoresis and transferred to nitrocellulose membranes. The membranes were blocked with 5% skimmed milk for 1 h and then incubated overnight with specific primary antibodies at 4°C. The following antibodies were used: anti-DLX3 (code: sc-18143, Santa Cruz, CA, USA), anti-amelogenin (kindly provided by Dr. DenBesten, University of California, USA), anti-enamelin (prepared as previously described) [22], and anti-GAPDH (code: TA-08, ZSGB-BIO). The membranes were then incubated with secondary sheep anti-mouse or sheep anti-rabbit horseradish peroxidase-conjugated linked antibodies (Santa Cruz) for 1 h. The protein signals were visualized with Chemiluminescent Substrate (Millipore, MA, USA) and exposed by the ChemiDoc XRS System (BioRad, CA, USA). GAPDH (glyceraldehyde-3-phosphate dehydrogenase) served as an internal control. The densitometric analysis was performed on grayscale images from 3 independent experiments with Multi-gauge software (Fuji, Japan).

### Real-time RT-PCR

Total RNA was isolated from LS8 cell cultures or mouse tooth germs using Trizol reagent (Vigorous Biotech), according to the manufacturer’s instructions. cDNA synthesis was carried out in a 25 μl reaction mixture containing 2 μg total RNA, 400 mM reverse transcription primers, 4 U/μl M-MLV, 1 U/μl RNAsin, and 0.4 mM dNTP mix, using M-MLV reverse transcriptase (Promega). The amplification reaction was carried out in an ABI 7300 Real-Time PCR System (Applied Biosystems, CA, USA) with SYBR Green Master Mix (Toyobo, Osaka, Japan) and the appropriate primers (Table 1). The annealing temperature was 60°C. Transcription levels were normalized against GAPDH, and each value is the average of three independent experiments.

**Table 1:** RT-PCR primers.

Gene	Primer sequence	Species	GenBank accession number	PCR product size (bp)
*Enam*	S 5’-TGGCAATGGACTTTACCCCTATC-3’	Mouse	NM_017468.3	273
	AS 5’-GCATCAGGCACAGTTGAGTTTGTAG-3’			
*Amelx*	S 5’-TGAGGTGCTTACCCCTTTGAAGTG-3’	Mouse	NM_009666.3	216
	AS 5’-GGAACTGGCATCATTGGTTGC-3’			
*Ambn*	S 5’-TTGAGCCTTGAGACAATGAGAC-3’	Mouse	NM_009664.1	114
	AS 5-’AAGTCCGTGCAACCATAAACTAT-3’			
*Tuft1*	S 5’-CGGAACTGGTGTACCTTGGTG-3’	Mouse	NM_011656.2	152
	AS 5’-GGCATCATGGCATAGGTCTTC-3’			
*Mmp20*	S 5’-TACGAAGTGGCTGAACGAG-3’	Mouse	NM_013903.2	114
	AS 5’-TGGGAACCCGAAGTCATA-3’			
*Klk4*	S 5’-TTGCAAACGATCTCATGCTC-3’	Mouse	NM_019928.1	228
	AS 5’-TGAGGTGGTACACAGGGTCA-3’			
*Odam*	S 5’-GTCACATCCTCACCACAGCA-3’	Mouse	NM_027128.2	160
	AS 5’-GAGTTTCTGGAGCTGTGCCT-3’			
*Amtn*	S 5’-GGACCACTGAATGGACAGCA-3’	Mouse	NM_027793.1	191
	AS 5’-TCTGGTTTAGTGCCTGCCTG-3’			
*DLX3*	S 5’-AGCCCAGTATCTGGCCTTG-3’	Mouse	NM_010055.3	133
	AS 5’-CGGCACCTCCCCATTCTTA-3’			
*Gapdh*	S 5’- CCAGCCTCGTCCCGTAGACA-3’	Mouse	NM_008084.2	189
	AS 5’- CCGTTGAATTTGCCGTGAGT-3’			

As, antisense; S, sense.

### Chromatin immunoprecipitation assay

LS8 cells were transfected with pCI-neo-V5DLX3^WT^ for 48 h. Cells were cross-linked with 1% (v/v) formaldehyde at 37°C for 10 min. DNA was sheared by sonication and immunoprecipitated with nonspecific IgG or anti-DLX3 antibody (code: ab66390, Abcam) for 12 h at 4°C. Immune complexes were incubated with Protein A/G-Sepharose CL-4B (Amersham Biosciences, Uppsala, Sweden) for 2 h at 4°C. Protein-DNA cross-linking was reversed by overnight incubation at 65°C. The precipitated DNA was amplified by real-time PCR for fragments of the specific enhancer region. PCR products were separated onto a 2% agarose gel, visualized, and analyzed with a GelDoc-It TS Imaging System (UVP, Upland, CA, USA). The PCR primers used for chromatin immunoprecipitation (ChIP) assay are listed in Table 2.

**Table 2:** Primers for ChIP assay.

Fragments	Predicted binding site	Primer sequences (5’-3’)	Product size (bp)
*Enam-E1*	-4842/-4824 bp	S 5’-AAGAATGTATCAGTGGTTGG-3’	183
		AS 5’-GTTAAGCCTCAGTTTCCTCA-3’	
*Enam-E2*	-4650/-4632 bp	S 5’-TGAGGAAACTGAGGCTTAAC-3’	123
		AS 5’-TATTTATGGTGTCTTCGGAT-3’	
*Amelx-E1*	-5278/-5260 bp	S 5’-TCCATGGGGACATTGCATTT-3’	166
		AS 5’-ACACCTCAAATCTCAACCTTTCT-3’	
*Amelx-E2*	-1146/-1128 bp	S 5’-TCTTTGTGCCATCTACACCA-3’	160
		AS 5’-CAAATCTGGCTCCCAAAAGGC-3’	
*Klk4-E1*	-3711/-3693 bp	S 5’-AGCTACATCCCTCCAGCTTCA-3’	197
		AS 5’-ACAGTCTTCCCGACATGCTTC-3’	
*Odam-E1*	-4492/-4474 bp	S 5’-TCTGTGAGCCTCTTGGTGGAT-3’	180
		AS 5’-CGTTCATTCACCAGCACAAAAC-3’	
*Odam-E2*	-1769/-1747 bp	S 5’-AGGGATTCCATTTGCTGCAC-3’	193
		AS 5’-AGGATCACAAGTATTCTGATGAAA-3’	

As, antisense; S, sense.

### Luciferase assays

LS8 cells were seeded into 24-well plates at 1×10^5^ per well, and cultivated until 60% confluence. Constructs of pGL3-promoter, pGL*Enam-E1*, pGL*Enam-E2*, pGL*Amelx-E2*, pGL*Klk4-E1*, pGL*Odam-E2*, or Mut pGL*Enam-E1*, Mut pGL*Amelx-E2*, and Mut pGL*Odam-E2* were separately co-transfected into LS8 cells with pCI-neo-V5DLX3^WT^, pCI-neo-V5DLX3^TDO^, or pCI-neo, using VigoFect reagent according to the manufacturer’s instructions. The total amount of DNA per well was kept constant using pcDNA3.1 plasmid. Luciferase activity was measured by a dual luciferase assay system (Vigorous) and normalized to *Renilla* luciferase activity. All experiments were performed in triplicate, and each was repeated three times.

### Statistical analysis

The data are presented as mean ± standard deviation (SD). Comparisons between two groups were performed on GraphPrism5 statistical software (GraphPad Software, USA), using Student’s *t*-test or one-way analysis of variance. A significant difference was noted when *P* <0.05.

## Results

### Spatial and temporal expression of *Dlx3* during mouse amelogenesis

**Fig 1:**
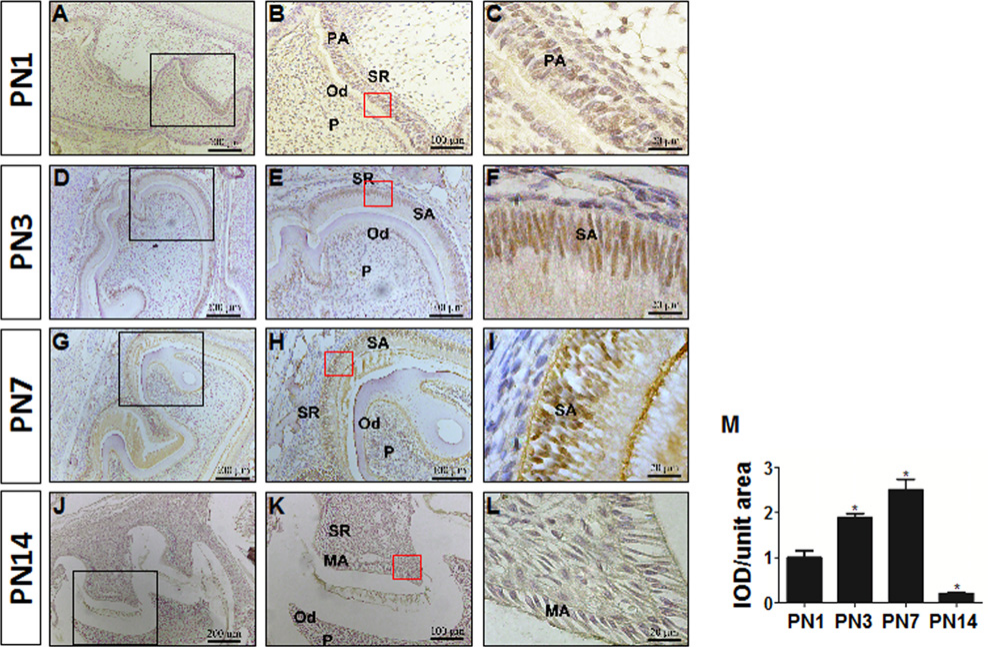
Immunostaining of DLX3 protein at different stages of mouse amelogenesis. (A-L) Immunostaining of DLX3 in sections of mouse molar germs at postnatal days 1 (A-C), 3 (D-F), 7 (G-I), and 14 (J-L). Representative figures from three independent experiments are shown. (M) The immunoreactivity of DLX3 protein within ameloblast cells was expressed as the mean optical density of the ameloblasts [integrated optical density (IOD)/unit area]. Each bar represents mean ± SD. *P <0.05 *vs*. the control (PN1 group). A, D, G, and J, original magnification ×100; B, E, H, and K, high-power magnification of the boxed areas in the low-magnification images, original magnification ×200; C, F, I, and L, high-power magnification of ameloblasts from the area inside the boxes in B, E, H, and K, original magnification ×1000. PA, pre-secretory ameloblasts; SA, secretory ameloblasts; MA, maturation ameloblasts; Od, odontoblast; P, dental pulp; SR, stellate reticulum layer. PN, postnatal day.

**Fig 2:**
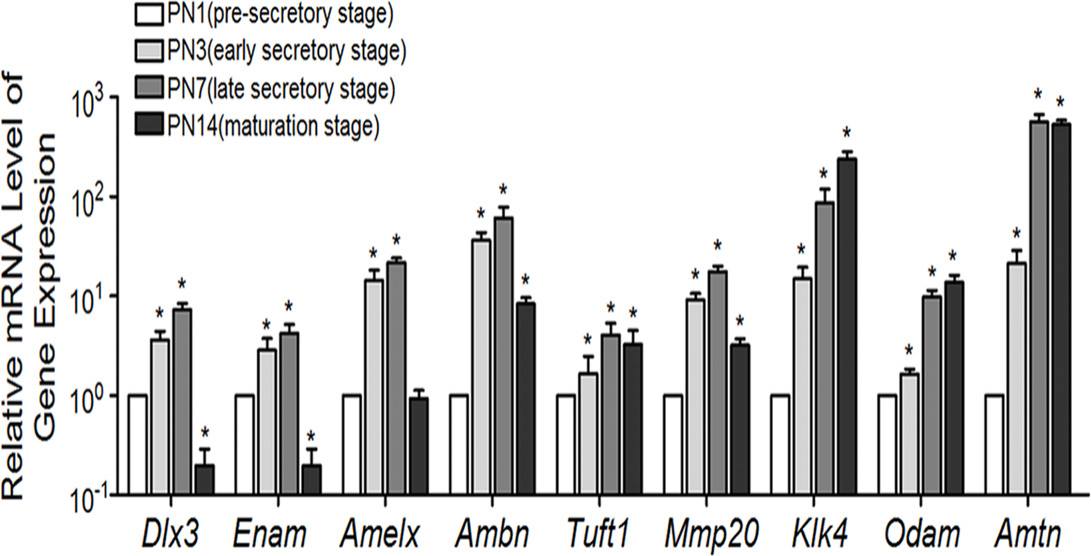
Expression patterns of *Dlx3* and EMP genes at the mRNA level during amelogenesis. Real-time RT-PCR was used to analyze the expression levels of *Dlx3* and EMP genes using RNA extracted from tooth germs at the indicated stages. The expression level at PN1 was set at 1, and the fold-changes at other stages were calculated relative to PN1. The data represent three independent experiments, and are shown as mean ± SD. *P <0.05 *vs*. the control (PN1 group). *Enam*, Enamelin; *Amelx*, Amelogenin; *Ambn*, ameloblastin; *Tuft*, Tuftelin-1; *Mmp20*, Matrix metalloproteinase 20; *Klk4*, kallikrein 4; *Odam*, Odontogenic ameloblast-associated protein; *Amtn*, Amelotin.

Immunostaining for DLX3 protein in mouse tooth germs of the mandibular first molar showed positive staining localized in the nuclei (Fig. 1A-L). The immunoreactivity of DLX3 staining was then quantified using Image-pro plus software (Fig. 1M). On postnatal day 1 (PN1), the enamel matrix had not been secreted in most area (except for the cusp region), indicating the pre-secretory stage of amelogenesis (Fig. 1A-C). DLX3 was weakly expressed in pre-secretory ameloblasts. At PN3, the enamel matrix was partially deposited and the cells indicated were entering the early secretory stage (Fig. 1D-F). Staining of DLX3 in early secretory ameloblasts was stronger than in pre-secretory ameloblasts. On PN7 sections, which represented the late secretory stage, DLX3 staining was even stronger in late than in early secretory ameloblasts (Fig. 1G-I). However, the staining of DLX3 significantly decreased and was hardly detectable in maturation-stage ameloblasts of PN14 mice (Fig. 1J-L). DLX3 staining was also observed in odontoblasts and dental pulp cells, but seldom in the stellate reticulum layer. Consistent with the expression pattern of DLX3 protein, the expression of *Dlx3* mRNA was elevated at the early secretory stage (PN3), further increased at late secretory stage (PN7), and then decreased at the maturation stage (PN14) (Fig. 2). The expression of all analyzed EMP genes *Enam*, *Amelx*, *Ambn*, *Tuft1*, *Mmp20*, *Klk4*, *Odam*, and *Amtn* was elevated at PN3, corresponding to the elevation of *Dlx3* expression (Fig. 2).

### Up- and down-regulation of enamel matrix protein genes *Enam*, *Amelx*, *Klk4*, and *Odam* by DLX3-overexpression and knockdown

**Fig 3:**
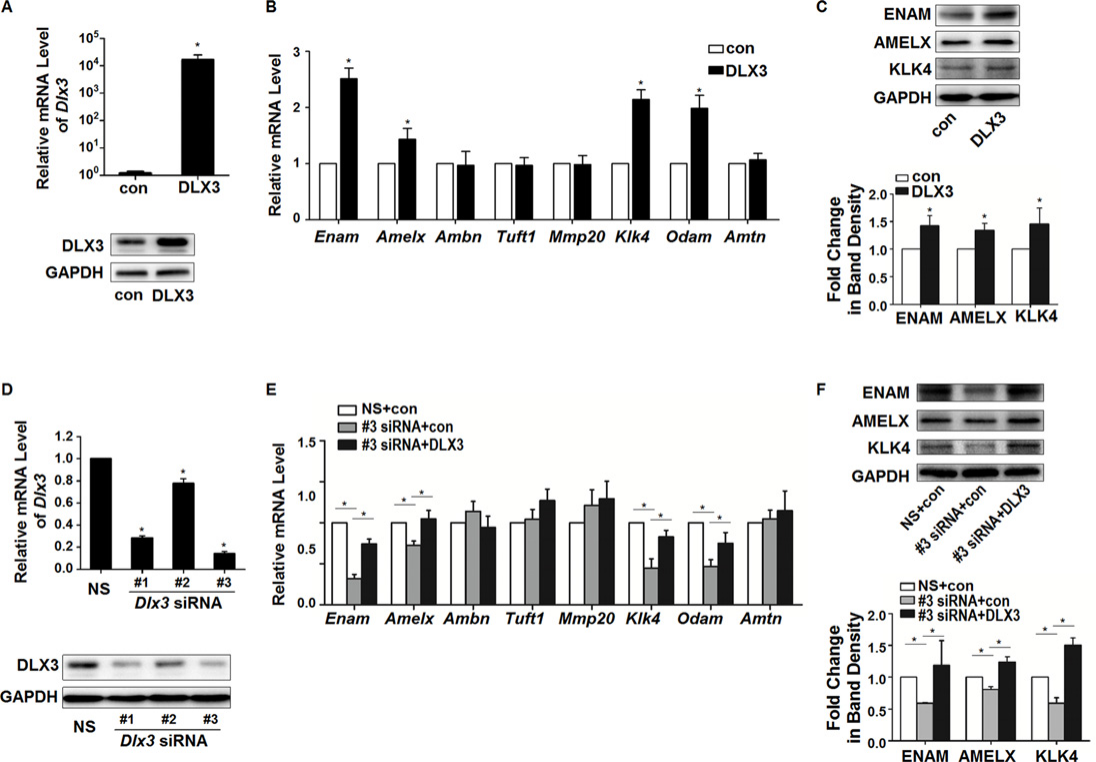
Regulation of *Enam*, *Amelx*, *Klk4*, and *Odam* by DLX3-overexpression and knockdown. (A) Over-expression of DLX3 was determined by real-time RT-PCR and western blot. Values are mean ± SD of the data from three independent experiments. *P <0.05 *vs*. control (con) group. (B) After 36 h of transfection, the expression of EMP genes was assessed. The mRNA expression of *Enam*, *Amelx*, *Klk4*, and *Odam* were significantly up-regulated. Values are presented as mean ± SD. *P <0.05 *vs*. the control (con) group. (C) After 48 h of transfection, elevated protein expression of ENAM, AMELX, and KLK4 were detected by western blot. Upper panel: western blot bands of ENAM, AMELX, and KLK4 (representative of three independent experiments). Lower panel: densitometric analysis of images of 3 independent experiments. *P <0.05 *vs*. the control (con) group. (D) Expression levels of DLX3 were determined after transfection with 3 independent *Dlx3*-specific small interfering RNAs (*Dlx3* siRNA #1, #2, #3), or a nonspecific siRNA, NS siRNA. Values are from three independent experiments, and shown as mean ± SD. *P <0.05 *vs*. NS group. (E) After DLX3 silencing (using *Dlx3* siRNA #3), the expression of EMP genes was assessed. The expression of *Enam*, *Amelx*, *Klk4*, and *Odam* was significantly down-regulated, and then successfully rescued by transfection of plasmid pCI-neo-V5DLX3^WT^. Values are mean ± SD of data from three independent experiments. *P <0.05. (F) At the protein level, expression of ENAM, AMELX, and KLK4 were also down-regulated by DLX3-knockdown and then rescued by pCI-neo-V5DLX3^WT^ transfection, as analyzed by western blot. Upper panel: western blot bands of ENAM, AMELX, and KLK4 (representative of three independent experiments). Lower panel: densitometric analysis of images of 3 independent experiments. *P <0.05.

After 24 or 36 h of DLX3^WT^ plasmid transfection, the over-expression efficiency of DLX3 mRNA and protein in LS8 cells was confirmed (Fig. 3A). With Dlx3-overexpression, 4 EMP genes, *Enam*, *Amelx*, *Klk4*, and *Odam*, were up-regulated by DLX3 at the mRNA level (Fig. 3B), compared with control. Western blot showed the corresponding up-regulation of ENAM, AMELX, and KLK4 proteins (Fig. 3C). Since there is no commercially-available antibody against mouse ODAM protein, it was not analyzed in this study.

Expression of DLX3 in LS8 cells was successfully knocked down by siRNA fragments specific for DLX3, particularly by *Dlx3* siRNA #1 and #3 (Fig. 3D). The most effective #3 *Dlx3* siRNA were then used for the subsequent knockdown experiments. After DLX3 silencing, *Enam*, *Amelx*, *Klk4*, and *Odam* were down-regulated compared with the NS group, consistent with the up-regulation effects of DLX3 over-expression (Fig. 3E). Western blot analysis revealed that the protein levels of ENAM, AMELX, and KLK4 were also decreased after DLX3 knockdown (Fig. 3F). The down-regulated expression of *Dlx3* and its targets genes, *Enam*, *Amelx*, *Klk4* and *Odam*, mediated by *Dlx3* siRNA, was then successfully rescued by transfection of plasmid pCI-neo-V5DLX3^WT^, as analyzed by qPCR and western blot, excluding the possibility of siRNA off-target effects (Fig. 3E and F).

### DLX3 directly transactivates the expression of *Enam*, *Amelx*, and *Odam* through binding to the enhancer regions

**Fig 4:**
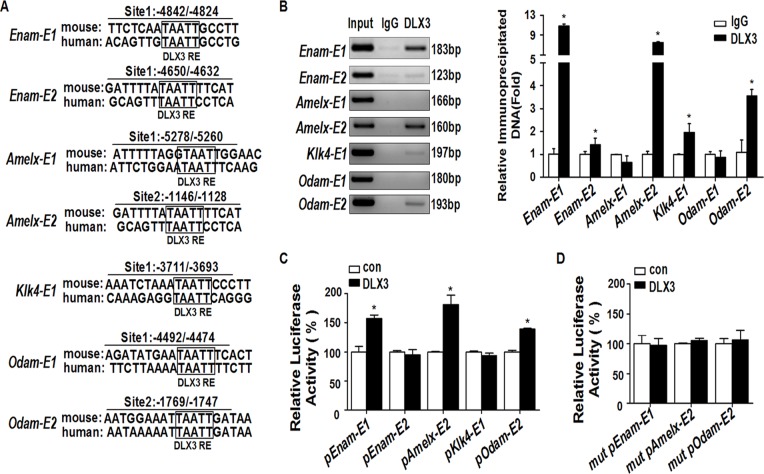
DLX3 directly transactivates the expression of *Enam*, *Amelx*, and *Odam* by binding to the enhancer regions. (A) Bioinformatic analysis was performed on 6000 bp 5’-flanking regions of *Enam*, *Amelx*, *Klk4*, and *Odam*. 1 or 2 conserved DLX3 response elements were separately found on the four genes (translation start site defined as +1 bp). RE, response element. (B) ChIP assays determined whether DLX3 was recruited to the predicted enhancer sites. Left panel: gel images of PCR products, representative of three independent experiments. Right panel: statistics of the PCR results. Values are from three independent experiments, and shown as mean ± SD. *P <0.05 *vs*. IgG group. (C) Luciferase assays were performed to evaluate the impact of DLX3 on the transcriptional activity of each constructed luciferase reporter, which contained enhancer sequences of specific potential DLX3 response elements. Data are compared with the control group (con), and presented as mean% ± SD. *P <0.05. *pEnam-E1* represents pGL*Enam-E1*, etc. (D) Mutations of DLX3 binding sites reduced the activation effect of DLX3 on the transcriptional activity of the pGL*Enam*-E1, pGL*Amelx*-E2, and pGL*Odam*-E2 reporters. The data represent the mean% ± SD of three independent experiments, each experiment performed in triplicate. *P <0.05 *vs*. the control (con) group. *mut pEnam-E1* represents Mut pGL*Enam-E1*, etc.

For *Enam*, *Amelx*, *Klk4*, and *Odam*, bioinformatic analysis was performed separately on 6000 bp 5’-flanking regions upstream of the translation start sites (defined as +1 bp) using MatInspector software. Conserved DLX3 potential binding sites were predicted on the enhancer regions of these 4 EMP genes, and the locations are named as follows: *Enam-E1* (representing the first potential DLX3 binding site on the *Enam* enhancer), -4842/-4824 bp; *Enam-E2*, -4650/-4632 bp; *Amelx-E1*, -5278/-5260 bp; *Amelx-E2*, -1146/-1128 bp; *Klk4-E1*, -3711/-3693 bp; *Odam-E1*, -4492/-4474 bp; and *Odam-E2*, -1769/-1747 bp (Fig. 4A). Further ChIP assays confirmed the recruitment of DLX3 onto the predicted binding sites of *Enam-E1*, *Enam-E2*, *Amelx-E2*, *Klk4-E1*, and *Odam-E2* (Fig. 4B). The primers for the distal region outside the 6000 bp 5’-flanking region of *Enam*, *Amelx*, *Klk4*, and *Odam* were designed and used as negative controls (S1 Table and S1 Fig.).

To characterize the functional significance of this DLX3 recruitment, luciferase assays were performed with pGL3-promoter luciferase reporters covering the binding site and the surrounding sequence of ~200 bp. The results showed the transcriptional activation of pGL*Enam-E1*, pGL*Amelx-E2*, and pGL*Odam-E2* by DLX3 recruitment, but not pGL*Enam-E2* and pGL*Klk4-E1*, compared with control (Fig. 4C). This DLX3-dependent activation of *Enam*, *Amelx*, or *Odam* was also dose-dependent (S2 Fig.). These activation effects were disrupted by mutations of the DLX3 binding sites on pGL*Enam-E1*, pGL*Amelx-E2*, and pGL*Odam-E2* (Fig. 4D).

### TDO mutation of DLX3 inhibits the activation effects of wild-type DLX3 on EMP genes

**Fig 5:**
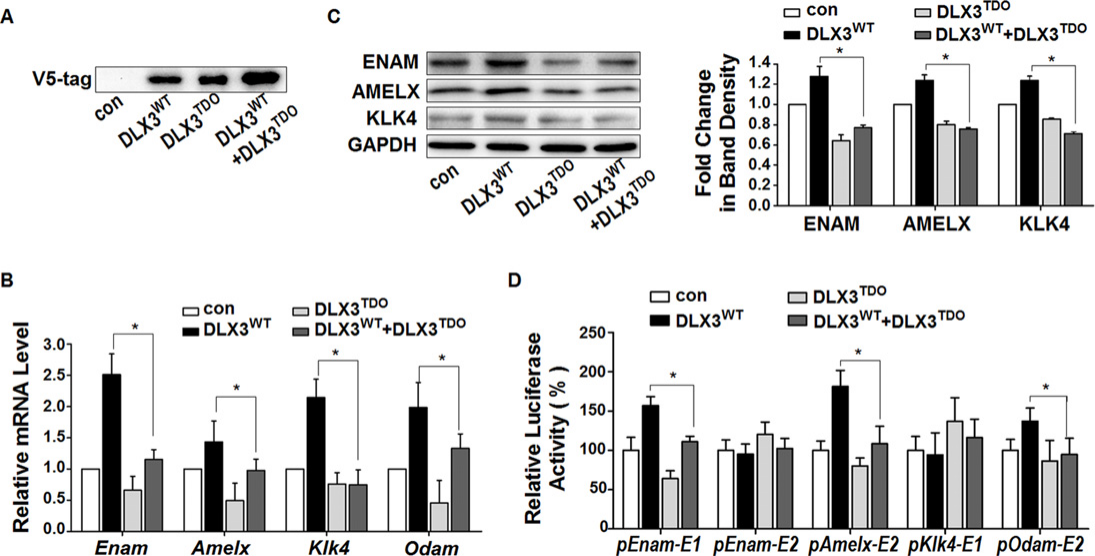
TDO mutation of DLX3 inhibits the activation of EMP genes by wild-type DLX3. (A) The plasmids pCI-neo (control empty vector, con), V5DLX3^WT^, and V5DLX3^TDO^ were transfected into LS8 cells. Equal amounts of V5DLX3^WT^ or V5DLX3^TDO^ plasmid were added to each group, and the total amounts of plasmid in the groups were kept constant using pCI-neo. Over-expression of V5DLX3^WT^ or/and V5DLX3^TDO^ were analyzed by western blot using antibody against V5-tag. A representative figure from three independent experiments is shown. (B) After over-expression, the mRNA expression levels of *Enam*, *Amelx*, *Klk4*, and *Odam* were evaluated. Values were obtained from three independent experiments, and are presented as mean ± SD. *P <0.05 between the DLX3^WT^ group and the DLX3^WT^+DLX3^TDO^ group. (C) Left panel: western blot for the protein expression levels of ENAM, AMELX, and KLK4 after transfection (representative of three independent experiments). Right panel: densitometric analysis of images of 3 independent experiments. *P <0.05 between the DLX3^WT^ group and the DLX3^WT^+DLX3^TDO^ group. (D) After co-transfection with pCI-neo (empty control vector), V5DLX3^WT^, V5DLX3^TDO^, or both V5DLX3^WT^ and V5DLX3^TDO^ plasmids, the relative transcriptional activity of the 5 reporter constructs containing potential DLX3 response elements were analyzed by luciferase assays. Data were compared with the control group (con), and are presented as mean% ± SD. *P <0.05 between the DLX3^WT^ group and the DLX3^WT^+DLX3^TDO^ group. *pEnam-E1* represents pGL*Enam-E1*, etc.

In osteoblast and keratinocyte cell lines, the mutated DLX3^TDO^ is known to have a dominant-negative effect on the transcriptional function of DLX3^WT^ [17,18]. To determine the influence of DLX3^TDO^ on the activating action of DLX3^WT^ in ameloblast-like cells, the V5DLX3^TDO^ plasmid was transfected alone or co-transfected with the V5DLX3^WT^ plasmid at a ratio of 1:1 into LS8 cells. The over-expression efficiency of V5DLX3^WT^ or/and V5DLX3^TDO^ was confirmed by western blot using an antibody against V5-tag (Fig. 5A).

In the presence of both DLX3^WT^ and DLX3^TDO^, the mRNA levels of *Enam*, *Amelx*, *Klk4*, and *Odam* were significantly reduced compared with DLX3^WT^ alone (Fig. 5B). Correspondingly, the protein levels of ENAM, AMELX, and KLK4 were down-regulated in the DLX3^WT^+DLX3^TDO^ group compared with the DLX3^WT^ group (Fig. 5C). Further luciferase assays revealed that the transcriptional activity of *Enam-E1*, *Amelx-E2*, and *Odam-E2* in the presence of both DLX3^WT^ and DLX3^TDO^ were decreased compared with the DLX3^WT^ group (Fig. 5D). These results indicated that in LS8 cells, DLX3^TDO^ inhibits the transcriptional activation of EMP genes by DLX3^WT^.

## Discussion

In the current study, we investigated the specific role of the transcriptional factor DLX3, and the expression patterns of *Dlx3* mRNA and protein during amelogenesis. Three EMP genes (*Enam*, *Amelx*, and *Odam*) were confirmed to be new direct targets of DLX3. The DLX3 mutant responsible for TDO interfered with the normal activation function of wild-type DLX3 in an ameloblast cell line, which partially explains the dental enamel defects in individuals with TDO.

Previous studies showed that DLX3 is expressed in mouse ameloblasts [9], yet the spatio-temporal expression of DLX3 in amelogenesis has not been reported. Our immunochemistry results revealed that DLX3 staining was strong in the nuclei of ameloblasts during secretory stages (PN3 and PN7), yet were relatively weak in the pre-secretory and maturation stages, consistent with the mRNA pattern. This expression pattern is in accord with the expression of EMP genes, suggesting us an association between DLX3 and EMP genes. Subsequent gain-of-function and loss-of-function studies identified that DLX3 promotes the expression of *Enam*, *Amelx*, *Klk4*, and *Odam* in ameloblast cell line, while mutant DLX3 inhibits this promoting function. Among these 4 EMP genes, *Amelx* and *Enam are both* associated with matrix apposition and mineralization, while *Odam* and *Klk4* are generally thought to participate in matrix mineralization [23]. Combined with the hypoplasia and hypomaturation types of amelogenesis imperfecta detected in TDO patients [3], we suggested that mutant DLX3 disrupts both the apposition and mineralization processes of amelogenesis through inhibiting the activation function of wild-type DLX3 on *Enam*, *Amelx*, *Klk4*, and *Odam*. DLX3 staining was also observed in odontoblasts, consistent with the regulation of *Dspp* (dentin sialophosphoprotein), one of the main dentin matrix protein genes, by DLX3 [16].

AMELX is the most abundant EMP expressed by secretory and early maturation ameloblasts, comprising >90% of the unmineralized enamel matrix, and is thought to form an organic scaffold that is essential for regulation of crystallite growth [24]. In humans, variety of mutations in *AMELX* gene are associated with enamel hypoplasia and/or hypomaturation (OMIM 301200) [25]. Recently, *in vitro* studies have demonstrated that another DLX member, DLX2, transactivates *Amelx* expression, yet the expression of DLX2 is very weak at the secretory stage and is elevated at the maturation stage [26]. Since our results showed the expression of DLX3 is high at the secretory stage and decreased at the maturation stage, it seems that the activation of *Amelx* expression at the secretory stage is mainly controlled by DLX3, and switches to DLX2 at the maturation stage.

ENAM, the largest EMP, is also expressed at the secretory and early-maturation stages, and is considered to participate in crystallite growth and elongation [27]. Clinically, multiple *ENAM* mutations cause smooth hypoplastic and local hypoplastic AI (OMIM 104500) with a grossly reduced enamel volume [28]. Transgenic studies found that the -5200~-3900 bp region of the *Enam* enhancer is related to its tissue-specific regulation [29], consistent with our finding that DLX3 transactivated *Enam* expression through binding to its -4842/-4824 bp enhancer (just between the -5200~-3900 bp region). This indicates DLX3 participates in the tissue-specific expression of *Enam* in ameloblasts.

ODAM is a structural protein considered to be crucial in enamel mineralization [30], no mutation of *ODAM* gene has been associated with AI till now. Our results showed that the expression of *Odam* remained at a high level in the late-maturation stage, yet *Dlx3* expression was very weak at this time. At different stages of osteoblast differentiation, the regulation of *RUNX2* and osteocalcin are co-regulated by the interaction and interplay between DLX3, DLX5, and MSX2 [14,31]. Thus, the regulation of *Odam* in amelogenesis at the late-maturation stage could be controlled by other transcription factors together with DLX3, for example, other DLX members, or MSX family members (another homeodomain family) [32].

KLK4 is one of the extracellular proteases that cleave structural proteins and function in enamel maturation and mineralization [33]. Mutations in *KLK4* gene are associated with pigmented hypomaturation AI (OMIM 204700) and the enamel is not fully mineralized [34]. Though the expression level of *Klk4* was affected by DLX3 over-expression or knockdown, the enhancer of *Klk4* was not activated by DLX3, suggesting *Klk4* maybe an indirect target of DLX3. The factor(s) mediating between DLX3 and *Klk4* remains to be clarified in further studies.

In addition to EMPs, interactions between epithelial ameloblasts and mesenchymal odontoblasts also affect the amelogenesis process. Disorders of odontoblast function inhibit the development of dental enamel, and *vice versa* [35,36]. In transgenic mice expressing DLX3^TDO^ under a Col1A1 promoter, DLX3^TDO^ that expressed in odontoblasts only (not ameloblasts) does not influence the enamel phenotype [15]. This suggests that the enamel disorders in TDO are caused by the specific influence of DLX3^TDO^ on amelogenesis, in line with our results that DLX3^TDO^ inhibited the activation of EMP genes by DLX3^WT^. Due to placental defects, DLX3-knockout mice die early, on embryonic day 9.5 [37]. To *in vivo* confirm the function of DLX3 in amelogenesis, conditional DLX3-knockout or DLX3-mutant models are being constructed for further studies.

In conclusion, we found that DLX3 up-regulates the EMP genes *Amelx*, *Enam*, *Odam*, and *Klk4* by directly binding to their enhancer regions or indirect mechanisms, while mutant-DLX3 (responsible for TDO) disrupts this regulatory function. Our results elucidate the specific function of DLX3 in amelogenesis and provide insights into the molecular mechanisms underlying the enamel defects in TDO disease.

## Supporting Information

S1 Data Raw values from the optical density analysis, densitometric analysis, Real-time PCR, and Luciferase assays in our study. (XLS)

S2 Data Raw values from the Western blot experiments in our study. (DOC)

S1 Fig Primers for the distal region outside the 6000 bp regulation regions of *Enam*, *Amelx*, *Klk4*, and *Odam* were designed and used as negative controls (NC) in ChIP assays. The data represent mean ± SD of three independent experiments, each performed in triplicate.
(TIF)

S2 Fig Titration experiment showing that DLX3 activated the specific enhancer regions of *Enam*, *Amelx* or *Odam* in a dose-dependent manner. The data represent mean ± SD of three independent experiments, each performed in triplicate.
(PNG)

S1 Table Primers for negative controls in the ChIP assay. As, antisense; S, sense.
(PNG)
