# Supplementary figures and images for: Transcriptional Factor DLX3 Promotes the Gene Expression of Enamel Matrix Proteins during Amelogenesis
Source: PLoS One. 2015 Mar 27;10(3):e0121288. doi: 10.1371/journal.pone.0121288 (PMC4376716; doi:10.1371/journal.pone.0121288)

Fig. 3A

Fig.3C
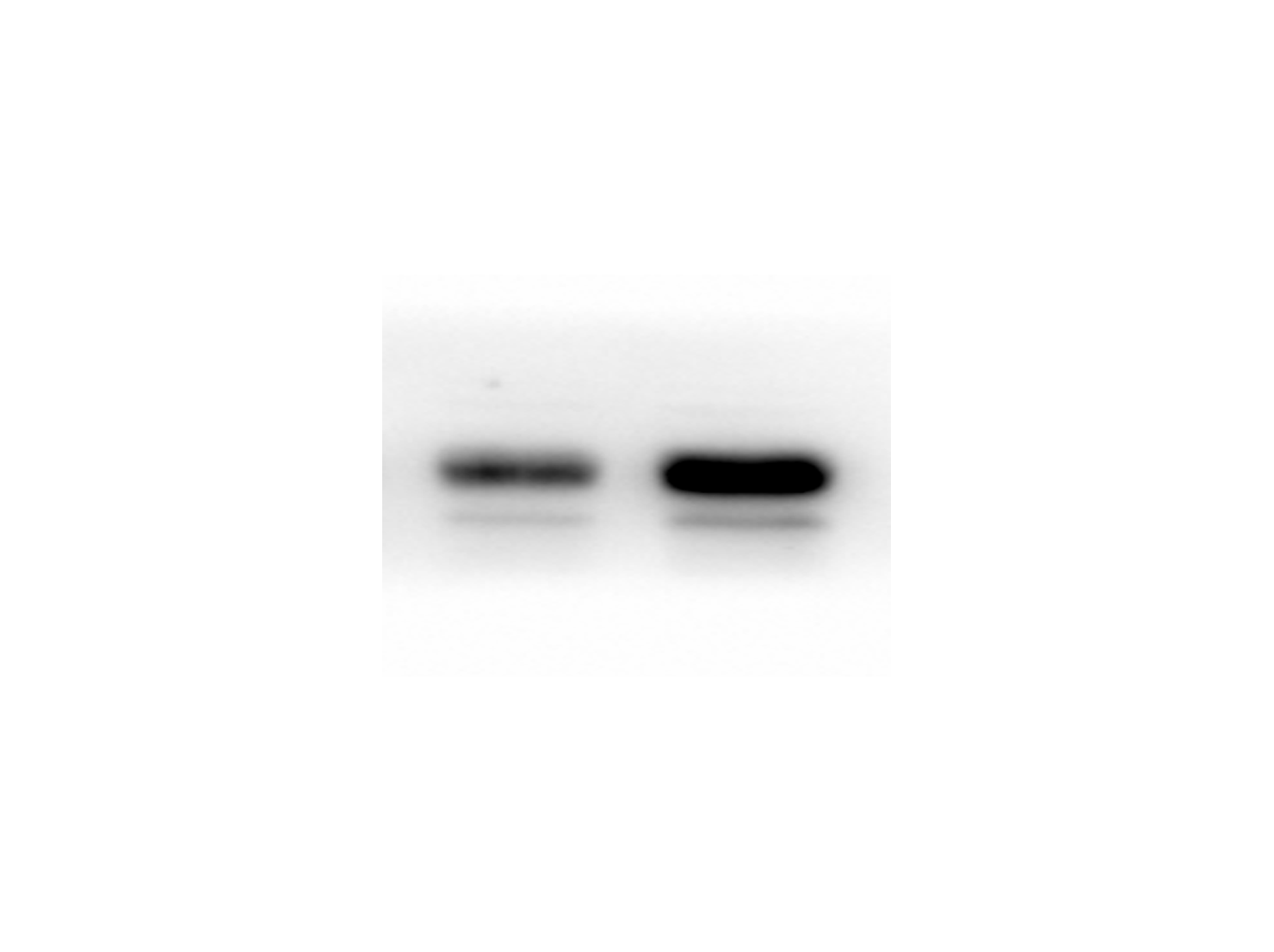

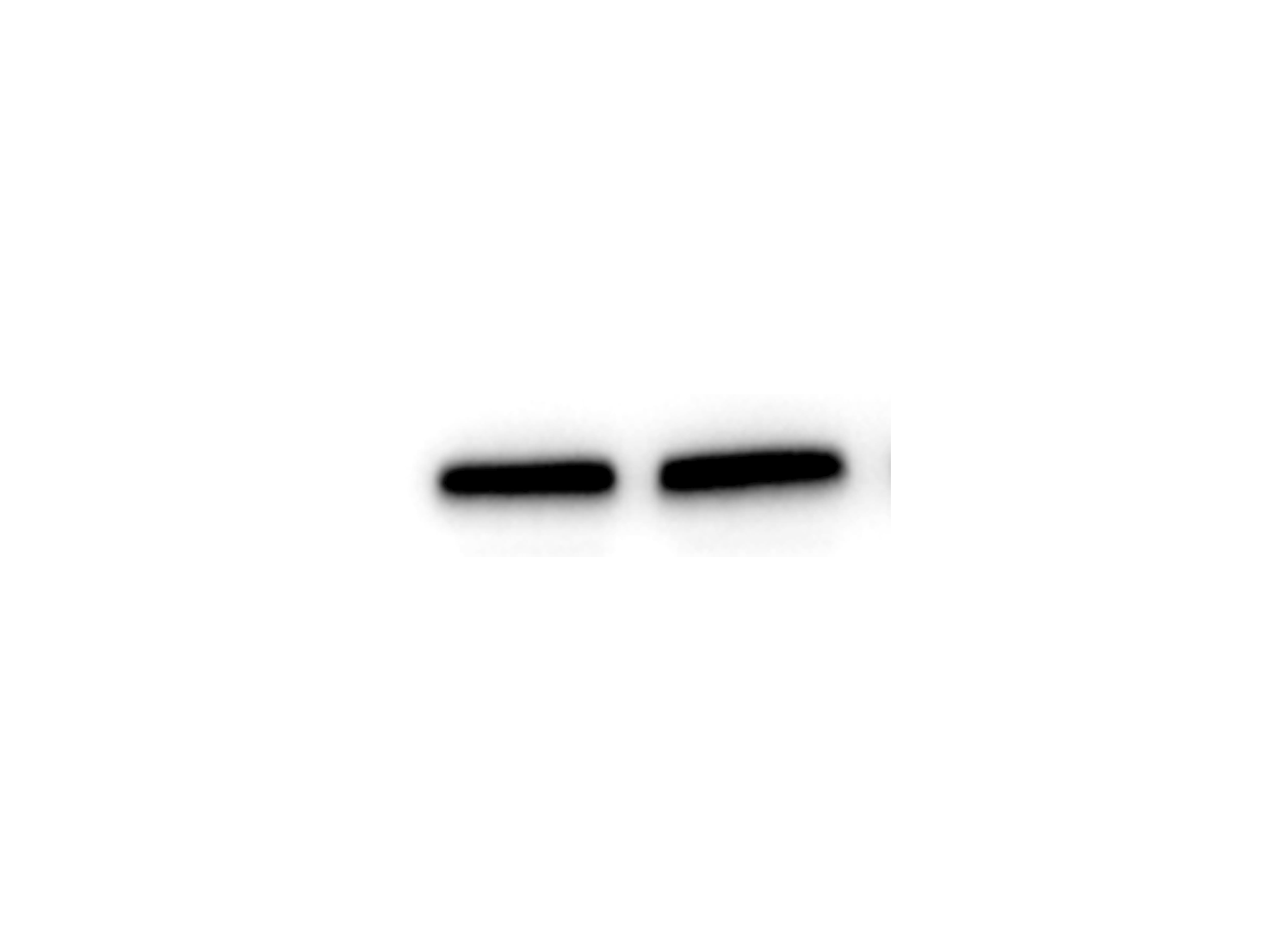


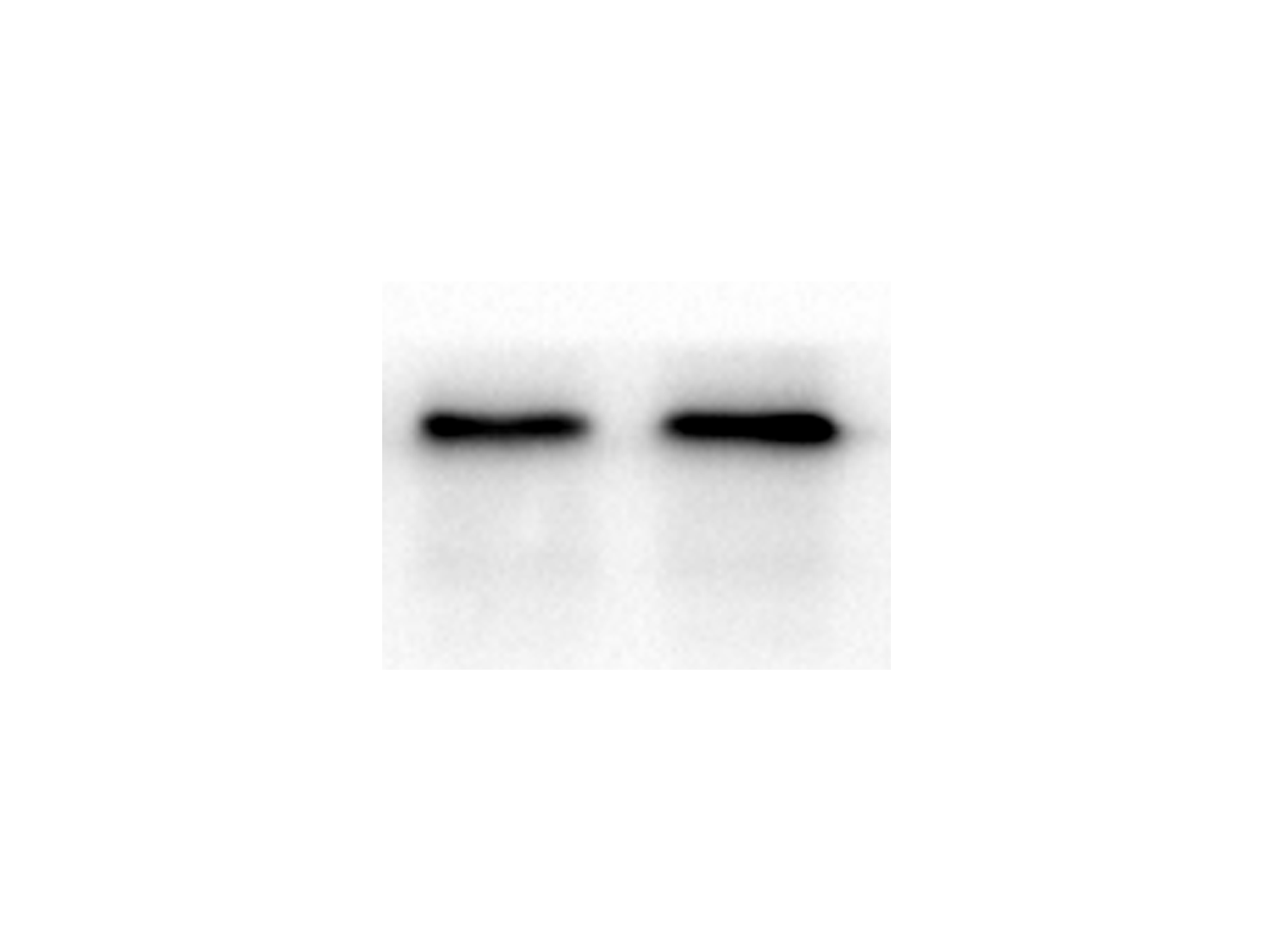

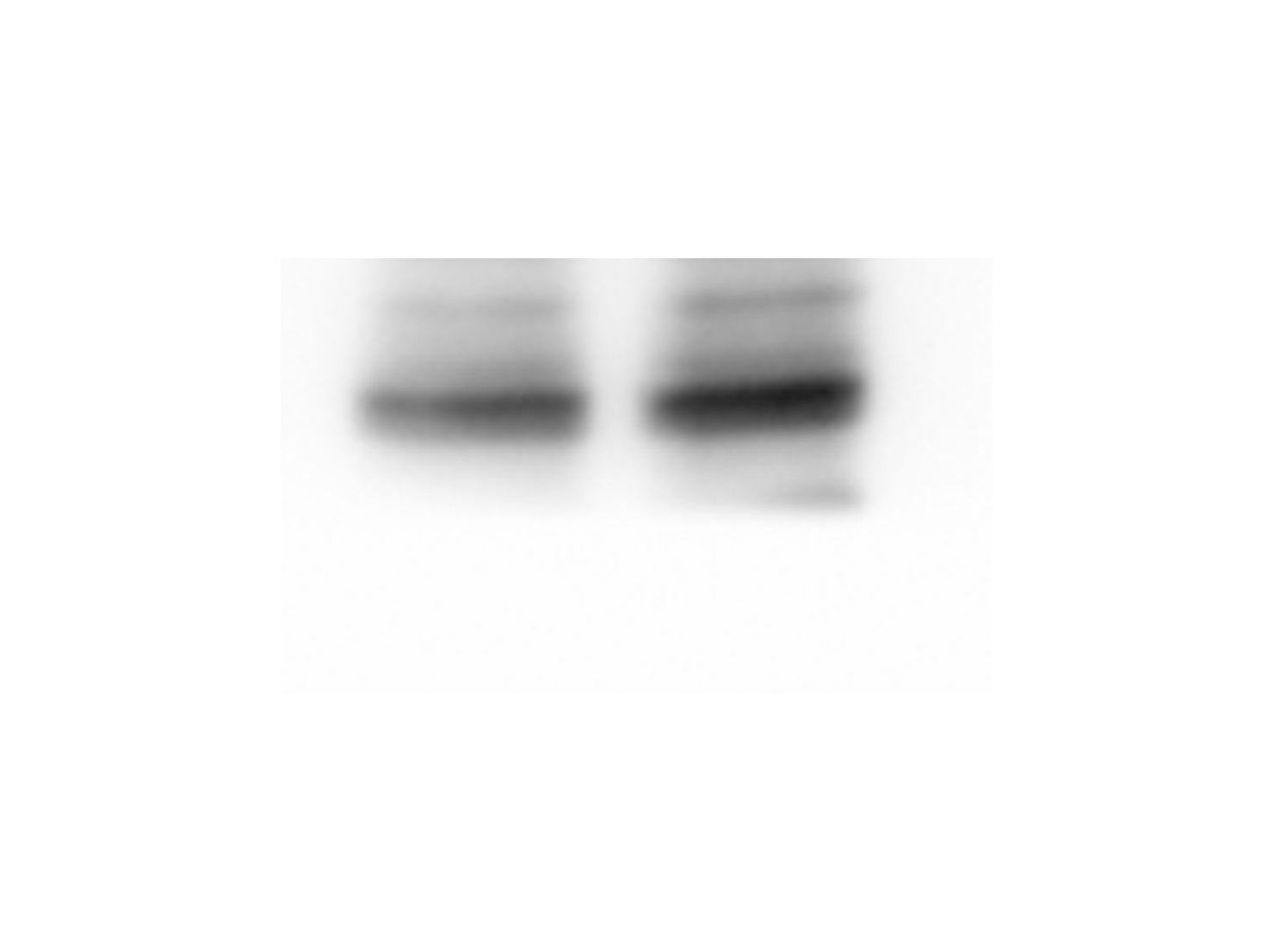


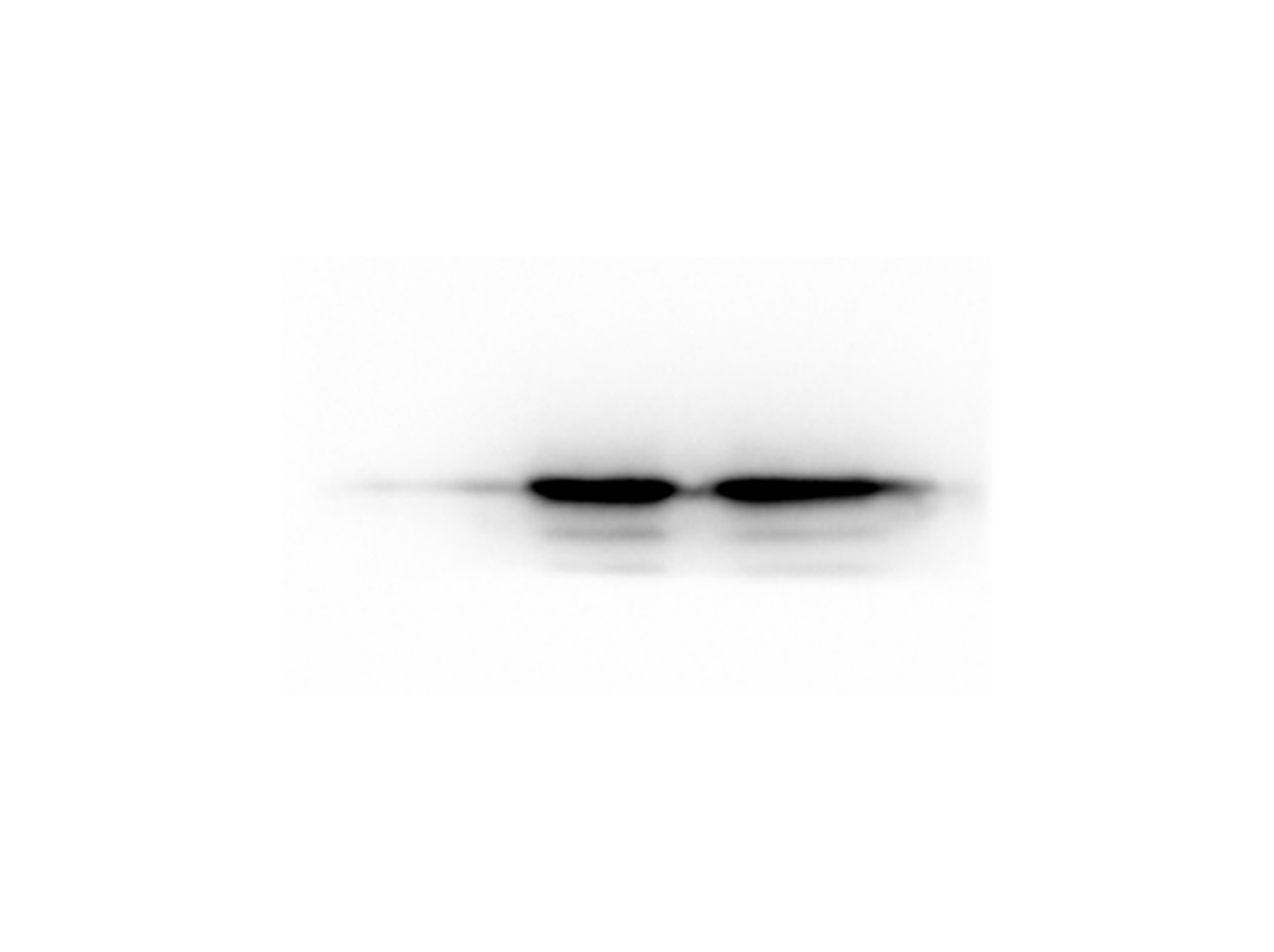

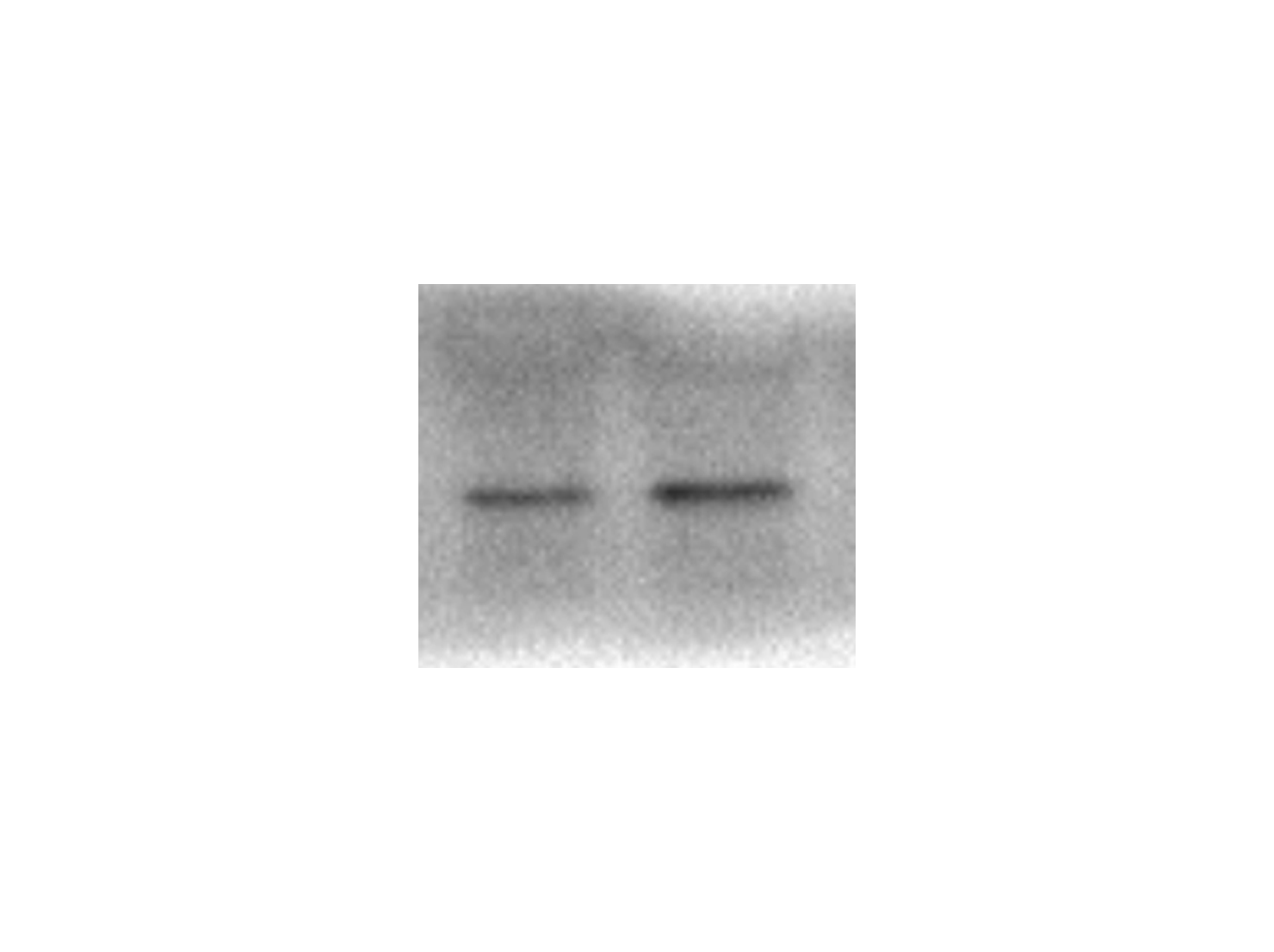


Fig. 3D


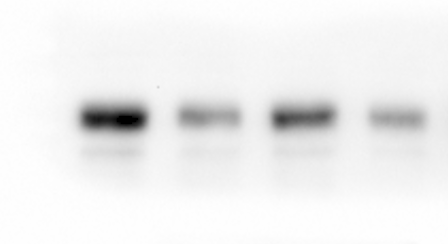

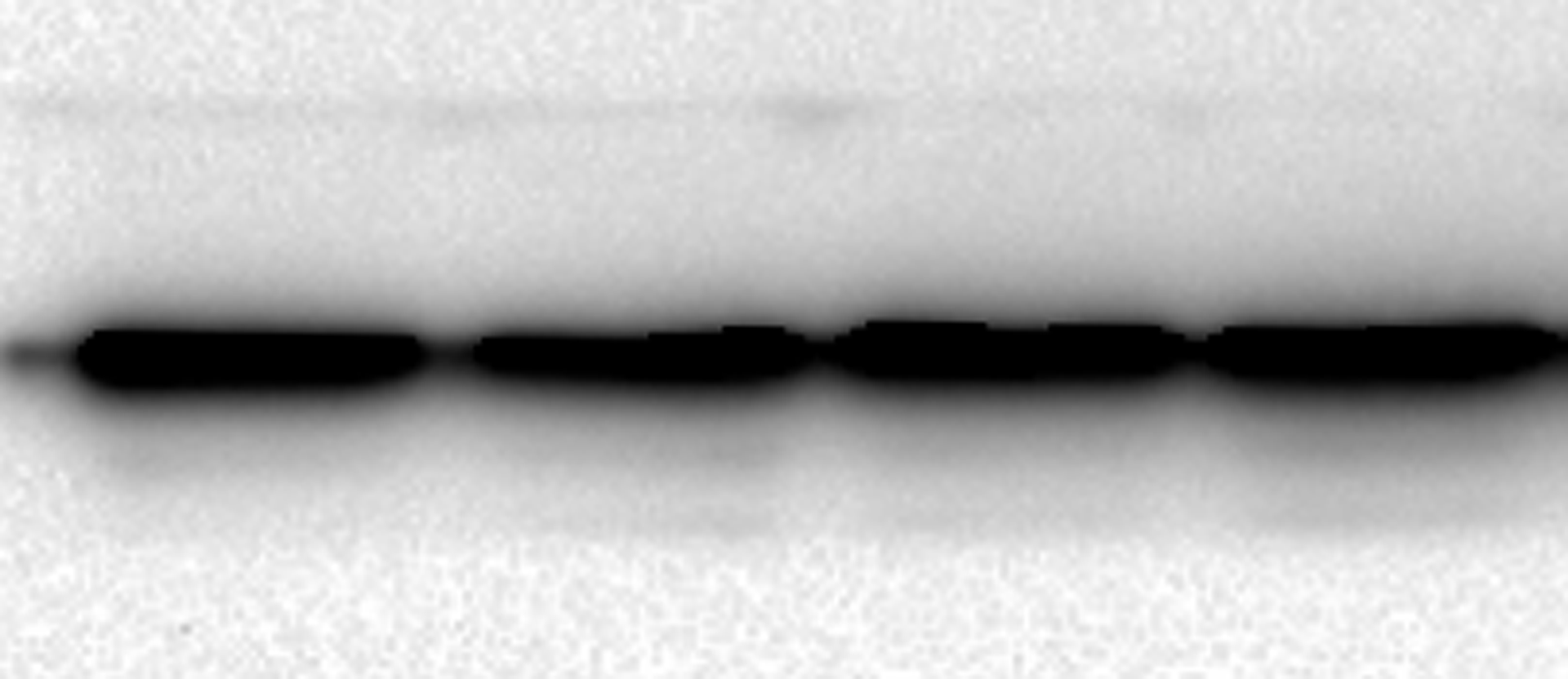


Fig. 3F


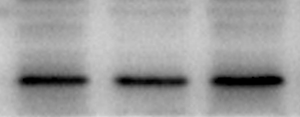


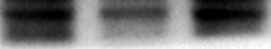


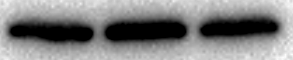


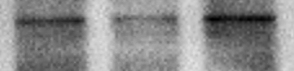


Fig. 4B


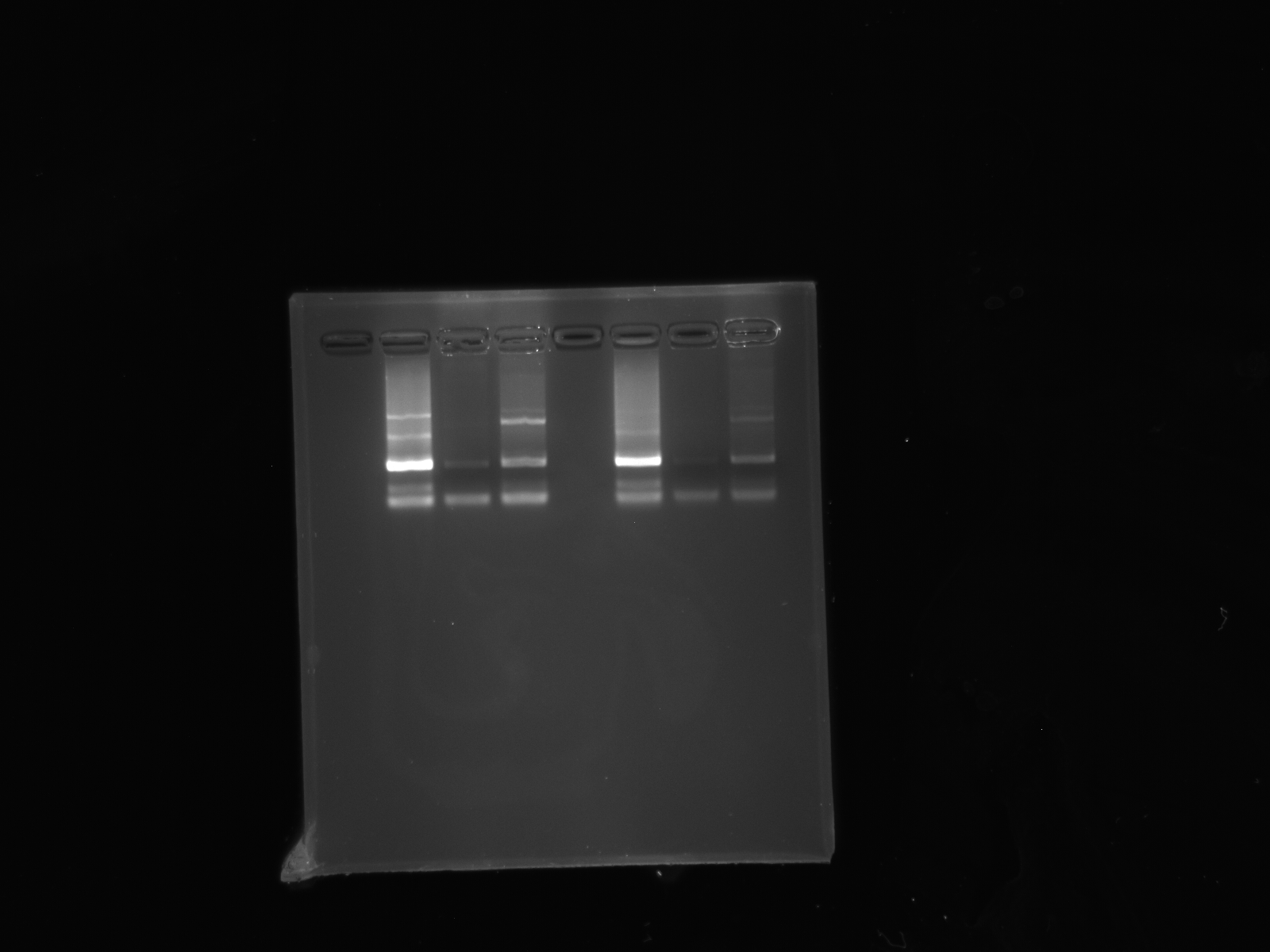

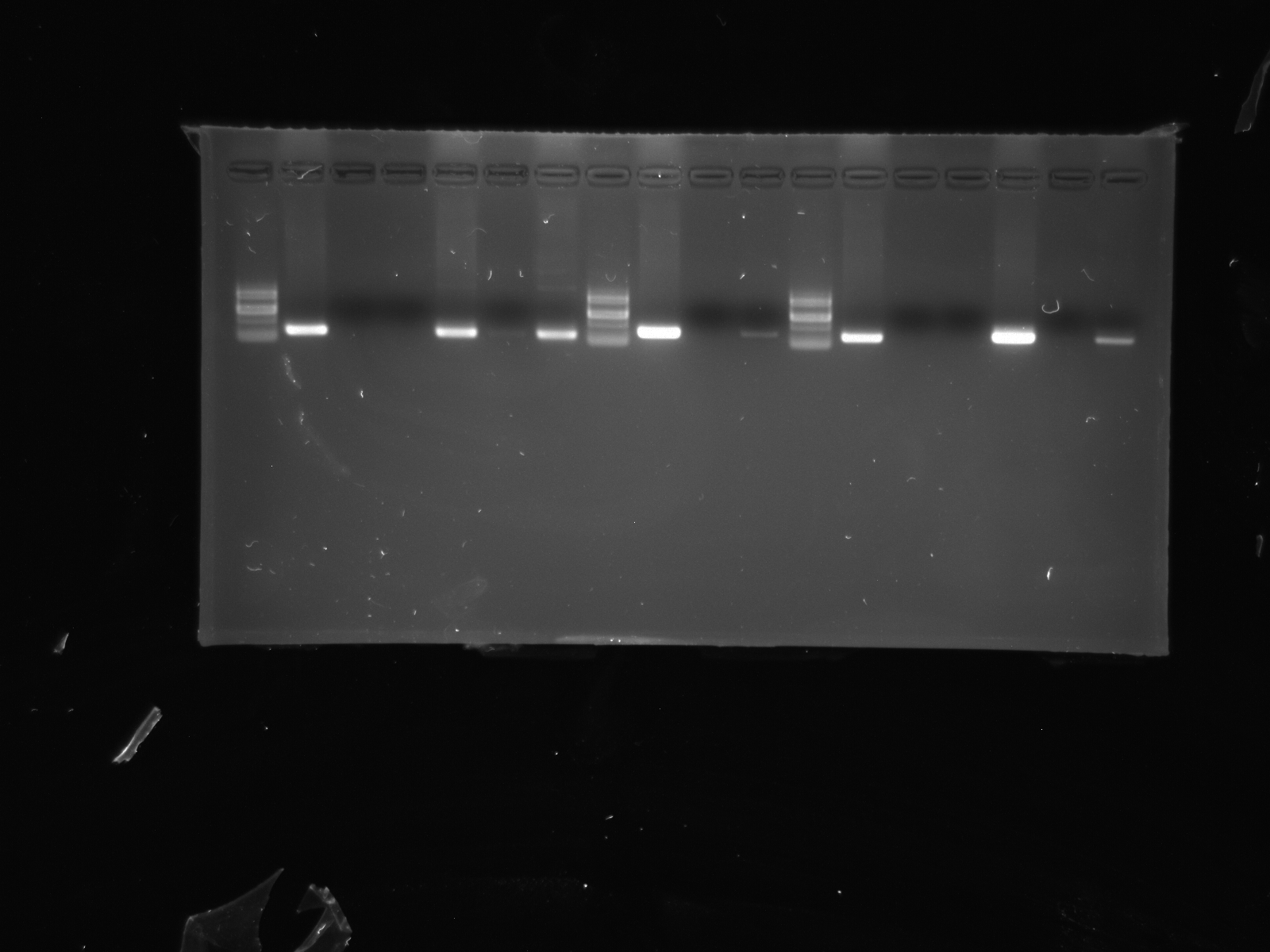


Fig.5A


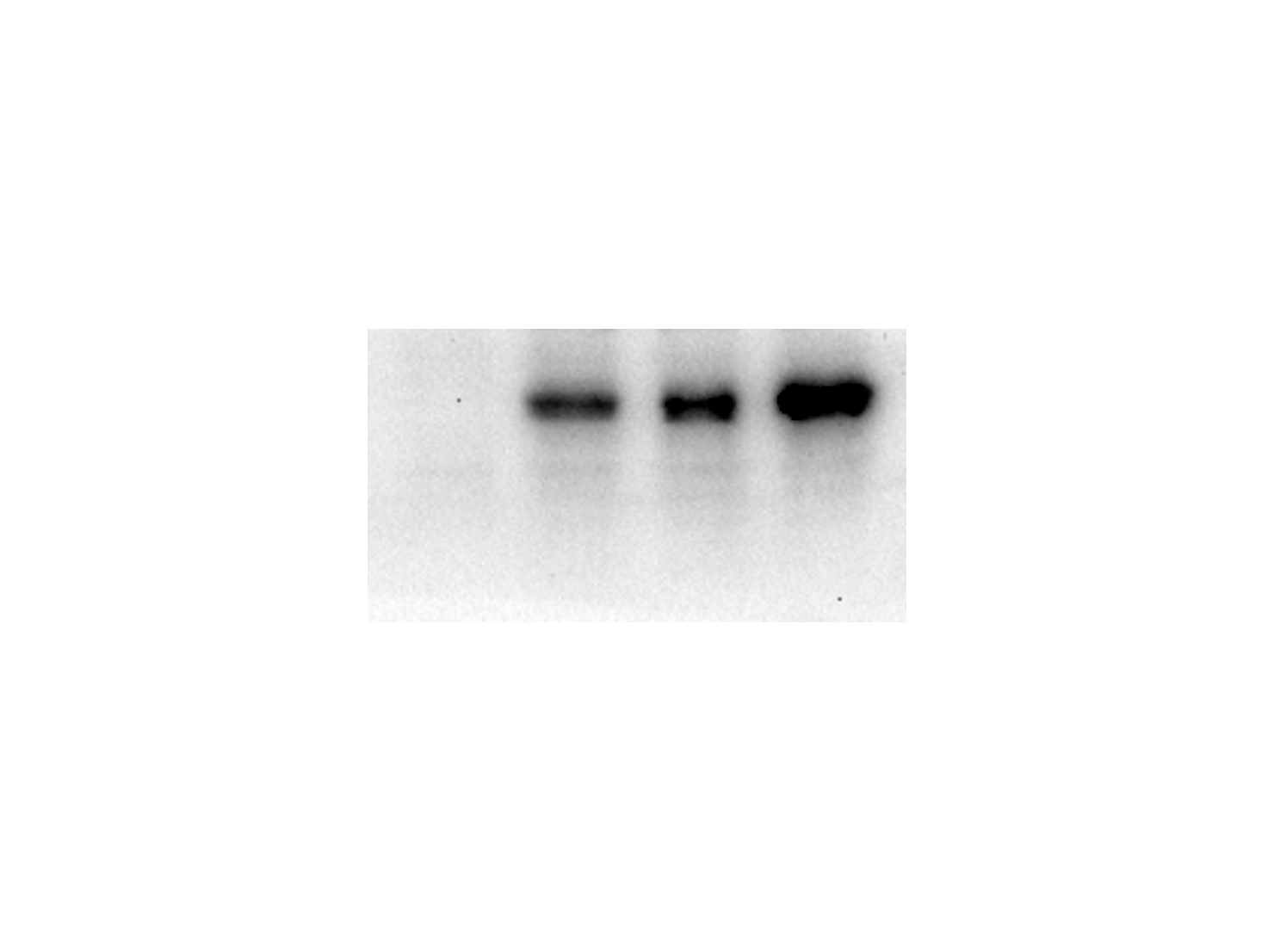


Fig.5C


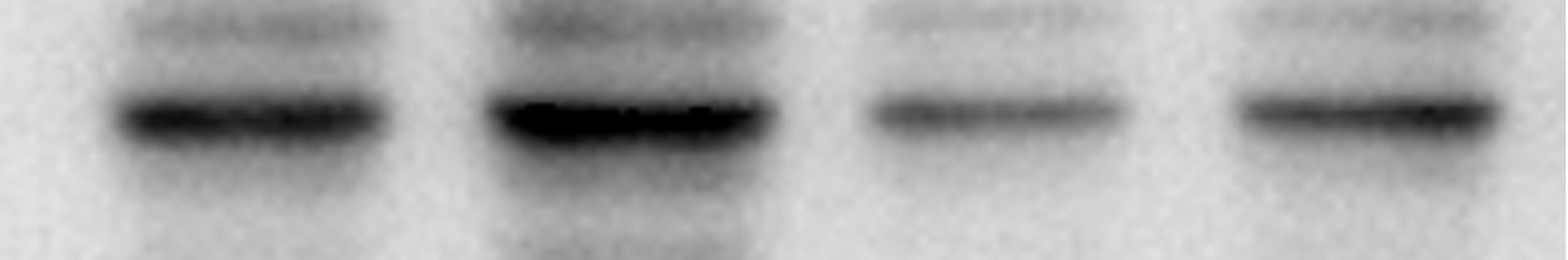

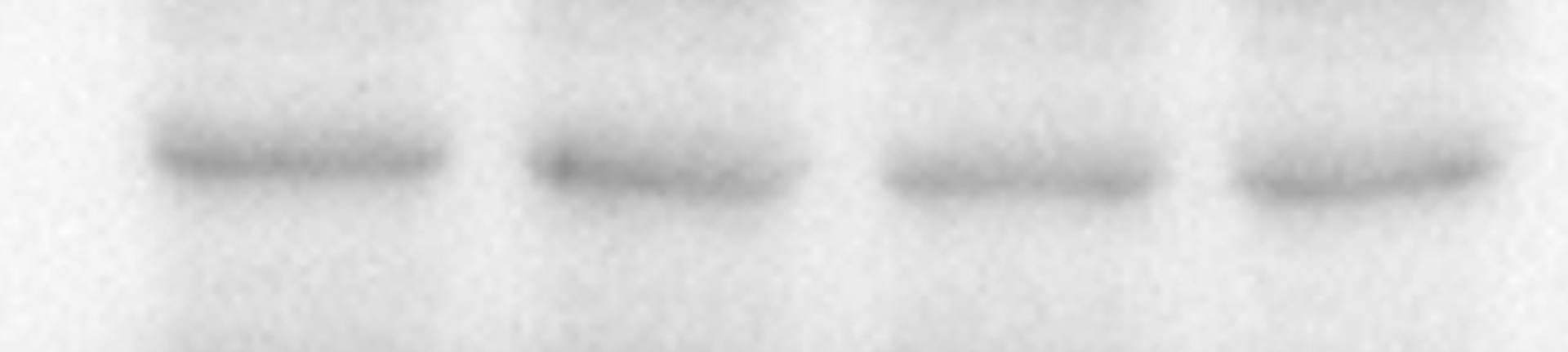

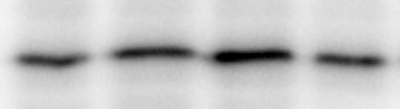


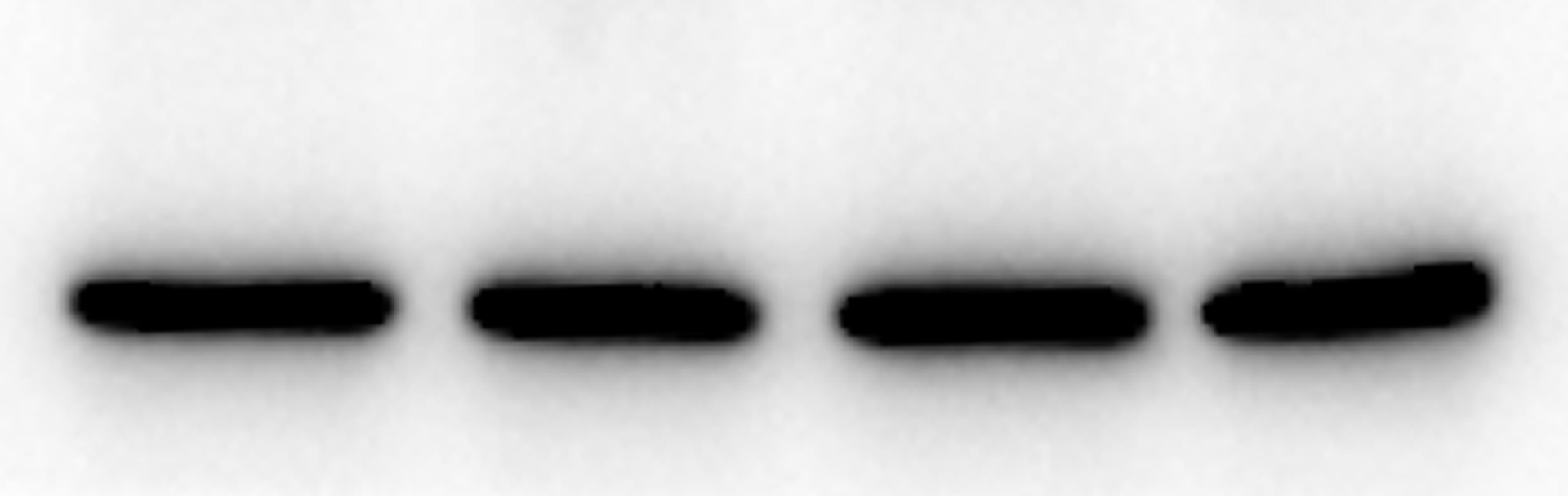

Supplement: S2 Data — (DOC) [file pone.0121288.s002.doc]

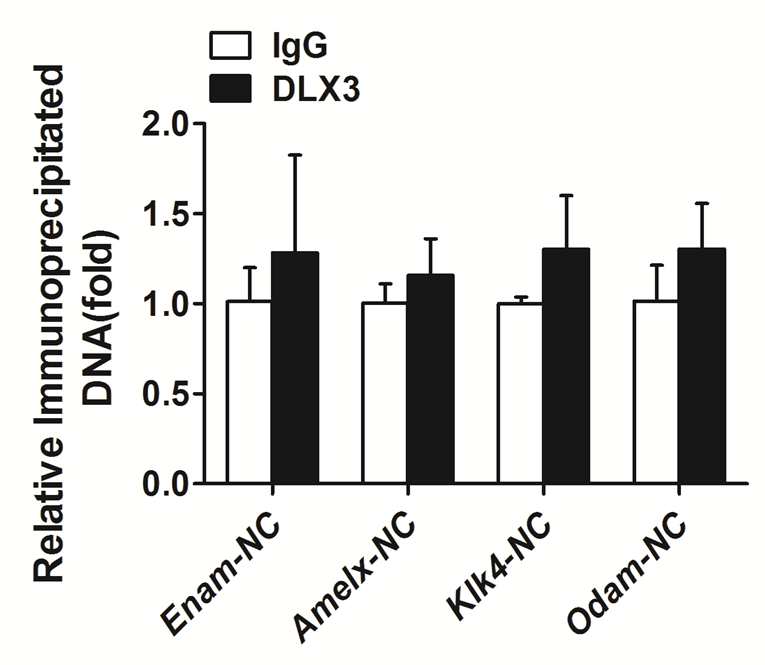

Supplement: S1 Fig — The data represent mean ± SD of three independent experiments, each performed in triplicate. (TIF) [file pone.0121288.s003.tif]

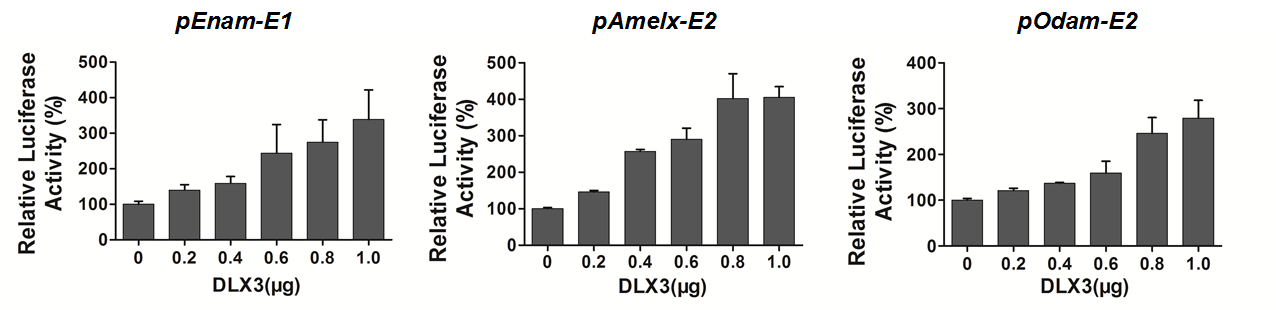

Supplement: S2 Fig — The data represent mean ± SD of three independent experiments, each performed in triplicate. (PNG) [file pone.0121288.s004.png]

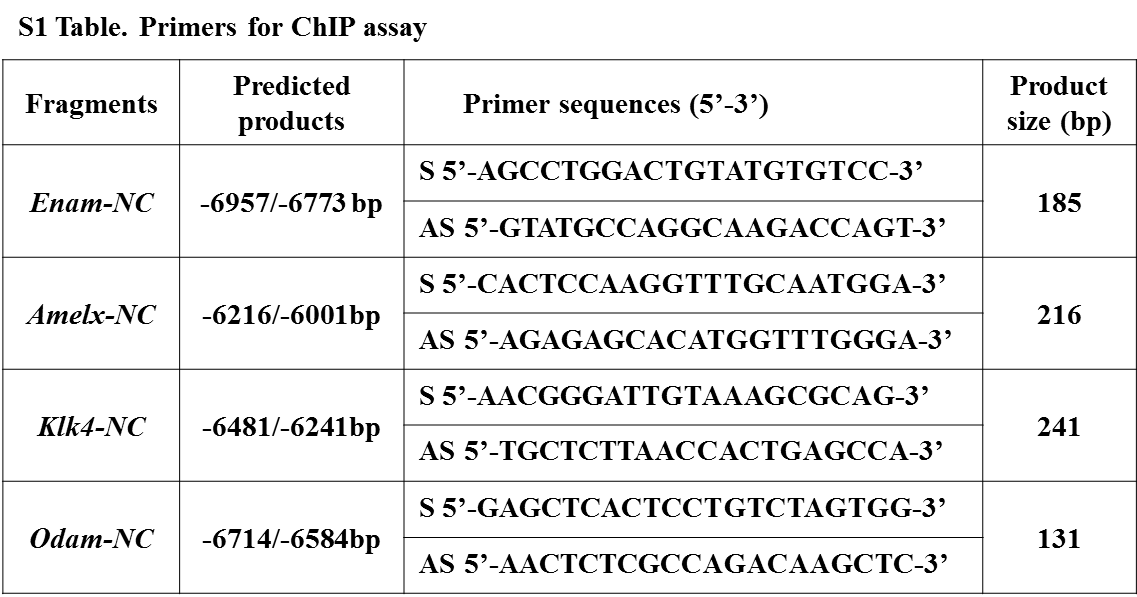

Supplement: S1 Table — As, antisense; S, sense. (PNG) [file pone.0121288.s005.png]
